# Coronary artery disease risk reclassification using an acoustic-based score in view of the new European Society of Cardiology 2019 guidelines on Chronic Coronary Syndromes

**DOI:** 10.1007/s10554-019-01746-y

**Published:** 2019-12-18

**Authors:** S. E. Schmidt, S. Winther, M. Boettcher

**Affiliations:** 1 Department of Health Science and Technology, Aalborg University, Aalborg, Denmark; 2 Department of Cardiology, Region Hospital Herning, Herning, Denmark

**Keywords:** Stable coronary artery disease, Heart sounds, Non-invasive testing, Reclassification, Cost-effectiveness

## Abstract

In August 2019, ESC published new guidelines on Chronic Coronary Syndromes including a new risk assessment paradigm for estimation of pre-test-probability. The CAD-score is an acoustic-based score for ruling-out of coronary artery disease (CAD). In the current letter to the editor we re-evaluate the re-classification potential the CAD-score in the view of the new guidelines.

## Introduction

In July 2019 the International Journal of Cardiovascular Imaging published our study about coronary artery disease (CAD) risk reclassification using an acoustic-based score [1]. A key point in the publication is the capability of the CAD-score to reclassify patient from their original pre-test-probability (PTP) to a lower risk class according to the 2013 European Society of Cardiology (ESC) guidelines on the management of stable CAD. In August 2019, ESC published their new guidelines on Chronic Coronary Syndromes [2]. Key changes in the 2019 guidelines related to estimation of PTP were that the threshold defining low risk was lowered from 15 to 5%, and the introduction of a new PTP score with PTP values considerably lower than the previously recommended score. In addition, the new score now includes dyspnea as a symptom. In the current letter to the editor we have recalculated the CAD-score reclassification outcome according to the new ESC 2019 guidelines.

## Methods

PTP was calculated for all patients referred with suspected CAD [1] using the new PTP table from the ESC 2019 guidelines. Patients with a PTP equal to or below 5% were classified as low likelihood. We reclassified patients with a PTP above 5% into the low likelihood group if their CAD-score was less or equal to 20. For two subjects, the new PTP score could not be calculated as they were below 30 years of age.

## Results

The mean PTP estimated from the ECS 2019 guidelines was 16.2% (SD: 11.5%) in the 1671 patients referred for testing due to suspected CAD. 178 (10.7%) had low likelihood of CAD (≤ 5%). Post CAD-score test this number increased to 679 (40.6%) (Fig. 2). Before testing, 6 (3.4%) patients with low PTP had significant-CAD, whereas post-reclassification this number increased to 28 (4.1%) (p = 0.52). If the PTP was not taken into consideration and all patients were classified only according to whenever their CAD-score was less or equal to 20, 631 patients (37.8%) were classified as low likelihood and 24 had significant-CAD (3.8%).

## Discussion

In this low prevalence population, the new PTP score from the ESC 2019 guidelines classified 10.7% of patients as low likelihood. Thereby, to avoid unnecessary testing, the need for reclassifying the remaining nearly 90% of patients, where the majority is not having CAD but non-invasive testing is recommended, is still evident. As in the original study, 4 out of 10 patients becomes classified as low risk after application of the CAD-score scheme. This reclassification did not increase the prevalence of CAD-subjects in the low risk group above the 5% limit recommended in the ESC 2019 guidelines (from 3.4 to 4.1%). Therefore, the relevance and effectives of the CAD-score as a potential rule-out device persists after application of the new ESC 2019 guidelines on Chronic Coronary Syndromes.
